# Data formats and standards for opportunistic rainfall sensors

**DOI:** 10.12688/openreseurope.16068.1

**Published:** 2023-10-10

**Authors:** Martin Fencl, Roberto Nebuloni, Jafet C. M. Andersson, Vojtech Bares, Nico Blettner, Greta Cazzaniga, Christian Chwala, Matteo Colli, Lotte de Vos, Abbas El Hachem, Charles Galdies, Filippo Giannetti, Maximilian Graf, Dror Jacoby, Hai Victor Habi, Petr Musil, Jonatan Ostrometzky, Giacomo Roversi, Fabiola Sapienza, Jochen Seidel, Anna Spackova, Remco van de Beek, Bas Walraven, Karina Wilgan, Xin Zheng

**Affiliations:** 1 Department of Hydraulics and Hydrology, Czech Technical University in Prague, Prague 6, 16629, Czech Republic; 2 IEIIT-CNR (National Research Council of Italy), Milano, Italy; 3 Swedish Meteorological and Hydrological Institute, Gothenburg, Sweden; 4 Institute of Meteorology and Climate Research, Campus Alpin, Karlsruhe Institute of Technology, Garmisch-Partenkirchen, Germany; 5 Chair of Regional Climate and Hydrology, Institute of Geography, University Augsburg, Augsburg, Germany; 6 Politecnico di Milano, Milan, Lombardy, Italy; 7 Artys srl, Genova, Italy; 8 Royal Netherlands Meteorological Institute, de Bilt, Netherlands Antilles; 9 Institute for Modelling Hydraulic and Environmental Systems, University of Stuttgart, Stuttgart, Germany; 10 Institute of Earth Systems, University of Malta, Msida, Malta; 11 Department of Information Engineering, University of Pisa, Pisa, Italy; 12 School of Electrical Engineering, Tel Aviv University, Tel Aviv-Yafo, Tel Aviv District, Israel; 13 Brno University of Technology, Brno, Czech Republic; 14 ISAC-CNR (National Research Council of Italy), Roma, Italy; 15 Department of Physics and Astronomy “Augusto Righi”, University of Bologna, Bologna, Italy; 16 Department of Water Management, Delft University of Technology, Delft, Netherlands Antilles; 17 GFZ Potsdam, Potsdam, Germany; 18 State Key Laboratory of Hydrology-Water Resources and Hydraulic Engineering, Center for Global Change and Water Cycle, Hohai University, Nanjing, Jiangsu, China

**Keywords:** opportunistic rainfall sensing, data standards, data format, naming conventions, commercial microwave links, satelllite microwave links, personal weather stations

## Abstract

Opportunistic sensors are increasingly used for rainfall measurement. However, their raw data are collected by a variety of systems that are often not primarily intended for rainfall monitoring, resulting in a plethora of different data formats and a lack of common standards. This hinders the sharing of opportunistic sensing (OS) data, their automated processing, and, at the end, their practical usage and integration into standard observation systems. This paper summarises the experiences of the more than 100 members of the OpenSense Cost Action involved in the OS of rainfall. We review the current practice of collecting and storing precipitation OS data and corresponding metadata, and propose new common guidelines describing the requirements on data and metadata collection, harmonising naming conventions, and defining human-readable and machine readable file formats for data and metadata storage.

We focus on three sensors identified by the OpenSense community as prominent representatives of the OS of precipitation:

- Commercial microwave links (CML): fixed point-to-point radio links mainly used as backhauling connections in telecommunication networks
- Satellite microwave links (SML): radio links between geostationary Earth orbit (GEO) satellites and ground user terminals.
- Personal weather stations (PWS): non-professional meteorological sensors owned by citizens.

The conventions presented in this paper are primarily designed for storing, handling, and sharing historical time series and do not consider specific requirements for using OS data in real time for operational purposes. The conventions are already now accepted by the ever growing OpenSense community and represent an important step towards automated processing of OS raw data and community development of joint OS software packages.

## Plain language summary

Opportunistic sensors, devices primarily intended not intended for sensing, are increasingly used for rainfall measurement. The lack of conventions defining which data should be stored and how, makes it difficult to automatically process the data and integrate these observations into standard monitoring networks. This paper reviews current practice of collecting and storing precipitation opportunistic sensing (OS) data based on the experience of more than 100 members of the OpenSense Cost Action and suggest common data format standards. We focus on three sensors identified by the OpenSense community as prominent representatives of the OS of precipitation: Commercial microwave links (CML), Satellite Microwave Links (SML), and Personal Weather Stations (PWS). The conventions are already now accepted by the ever growing OpenSense community and represent an important step towards automated processing of OS raw data and community development of joint OS software packages.

## Disclaimer

The views expressed in this article are those of the authors. Publication in Open Research Europe does not imply endorsement of the European Commission.

## Introduction

Characterising the spatial and temporal variability of rainfall represents a challenge in many areas where precipitation plays a key role, such as meteo-hydrology, climatology, weather forecasting, and water management ( Ahrens, 2012; Ochoa-Rodriguez *et al.*, 2015). Opportunistic sensing (OS) can complement standard precipitation measuring systems by crowd-sourced observations from personal weather stations or by sensors not primarily intended for precipitation monitoring, such as microwave wireless links. The number of opportunistic sensors has already exceeded the number of conventional instruments by an order of magnitude and is still increasing ( ECC, 2016; Lorenz & Kunstmann, 2012; Muller *et al.*, 2015; Tauro *et al.*, 2018). This paper focuses on three types of opportunistic sensors:

- Commercial microwave links (CML),
- satellite microwave links (SML),
- personal weather stations (PWS).

CMLs are point-to-point terrestrial radio links used as the backhaul of mobile networks. They operate at frequencies where radio waves are significantly attenuated by rain which enables their use as opportunistic rainfall sensors. SMLs are space-to-ground links (also, termed downlinks) between geostationary Earth orbit (GEO) telecommunication satellites (SatComs) providing one-way direct-to-home (DTH) TV broadcast or two-way broadband services ( *i.e.* satellite internet), and ground-based user terminals. Like CMLs, they operate at frequencies sensitive to raindrops and therefore they can be used for OS. Finally, PWS are non-professional meteorological sensors owned and operated by citizens. They are often equipped with rain gauges that directly measure rainfall. These three types of devices are extensively deployed worldwide and their potential for observing rainfall has been thoroughly investigated ( Bárdossy *et al.*, 2021; Chwala & Kustmann, 2019; Colli *et al.*, 2019; de Vos *et al.*, 2017; Giannetti & Reggiannini, 2021).

One of the major challenges of the OS is a lack of data standardisation. For instance, both CML and broadband services SML data are owned by private companies and generated for network monitoring purposes (let us notice that SML data can be freely collected by the users themselves in case of broadcast satellite downlinks). PWS data are collected by non-professional equipment of various manufacturers. These circumstances result in a plethora of different data standards and formats. In light of using OS data for research purposes, but also for future integration with datasets collected by conventional sensors, there is a strong need towards defining common data formats, standards and procedures for opportunistic rainfall sensors. This is one of the core objectives of the European COST Action OpenSense (CA20136) which was launched in 2021 to build a worldwide reference OS community.

In this paper, we review current OS data collection practices and formats as drawn from the experience of the more than 100 members of the OpenSense community and provide new guidelines which unify naming conventions, define mandatory, recommended and optional parameters to be stored, and propose netCDF as a common format for handling OS data. Moreover, we suggest a structure of netCDF files for CML, SML and PWS which define how to store data and metadata. Finally, we provide basic recommendations on how to store data in a non-binary (human-readable) form as CSV files. The review of data collection practices as well as the guidelines are based on an online survey performed within OpenSense Action, the personal experience of the authors, and discussions during dedicated joint OpenSense meetings of Working group on data management and standardization and Working group on method and software homogenization, which were held within the first and the second year of the Action, *i.e.,* in 2021 and 2022.

The paper is organised as follows. The section below describes the basic principles of operation of the three OS sensors considered here. Section Current practices in OS data collection overviews current practices in OS data collection. In section Proposed data and metadata standards we propose technical specifications for the information provided by opportunistic sensors. This section is followed by section recommending data formats, *i.e.*, how information should be structured, and finally, we conclude in the last section with the expected impact of the proposed guidelines.

## Opportunistic rainfall sensors

**Figure 1.**
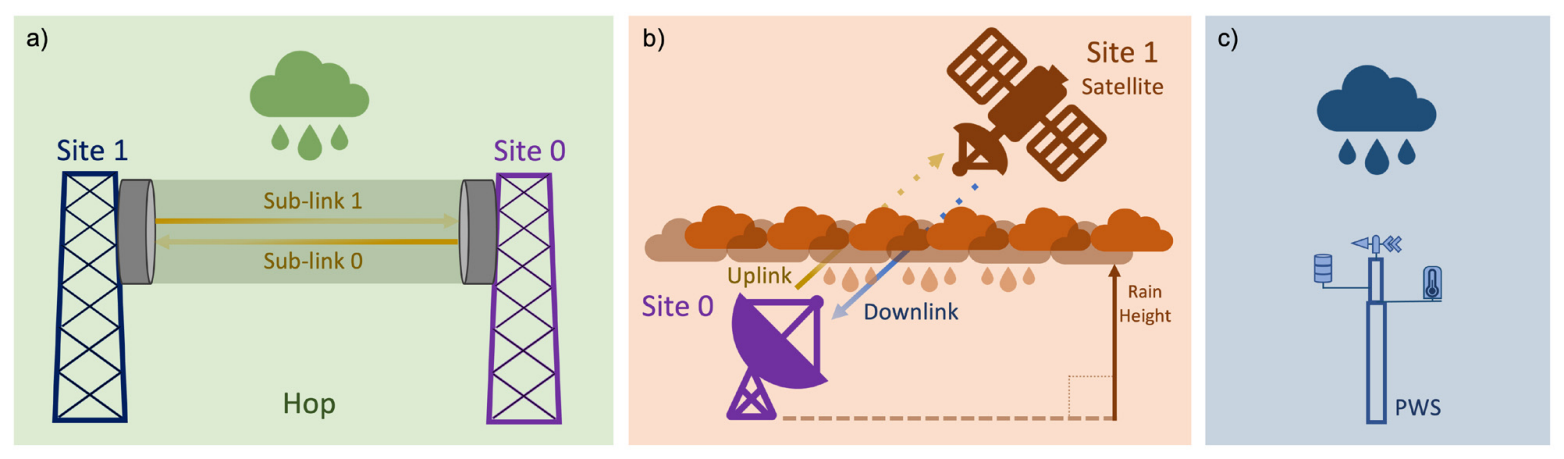
Sketch of the principle of operation of opportunistic rainfall sensors: **a**) commercial microwave links (CML), **b**) satellite microwave links (SML), and **c**) personal weather stations (PWS).

This section addresses the principles of CML, SML and PWS as rainfall sensors, as well as main features of the acquired data. An important difference between CML/SML and PWS is that microwave links carry out indirect measurements of rainfall, moving from electrical quantities. On the other hand, PWS are often equipped with simple rain gauges that collect raindrops and directly measure their volume. Figure 1a-c sketches how each of the three sensors work in detecting precipitation, whereas Table 1 summarises their main features.

**Table 1.** Main features of opportunistic rainfall sensors.

Sensor	Raw data	Spatial Integration	Domain size / location	Temporal Integration	Integration Time
**Commercial** **microwave links**	Received power level (RSL) and transmitted power level (TSL)	Straight line	From less than 100 m to few tens of km / tens of m above ground	Instantaneous values, averages or extremes	≤15 min
**Satellite** **microwave links**	RSL or SNR	Straight line (slant path)	Several km / near ground to rain height	Instantaneous values or averages	≤1 min
**Personal weather** **stations**	Rainfall depth (or rainfall intensity)	Single point Measurement	approx. 1 dm ^2^/ few metres above ground	Sum (or averages)	≥ 5 min

### Commercial microwave links

CMLs are fixed point-to-point line-of-sight radio links mainly used as backhauling links in telecommunication networks, *i.e.*, connecting distributed radio access nodes with the backbone of the network. Even though wireless backhauling links are being replaced with fibre optic in densely populated areas, the number of nodes is increasing due to network densification to support the innovative 5G technologies. Moreover, they remain dominant in many other contexts, covering large areas of land. CMLs are sometimes used to provide ‘last-mile’ internet connectivity or to provide building-to-building connectivity in LANs. Every CML provides bidirectional transmission between two radio units, that is, the two antennas in Figure 1a work as both transmitting and receiving terminals. Sometimes, dual frequency or multi-frequency links are used meaning that links transmit in each direction over several different frequency (or polarisation) channels ( ITU-R, 2017). In Figure 1 the term sublink is used to distinguish among all the possible signals travelling between the same pair of antennas.

CML have lengths of less than 100 metres up to tens of kilometres and most commonly use frequencies between 6 and 95 GHz ( ITU-R, 2017). At these frequencies, electromagnetic waves are scattered and absorbed by atmospheric particles. Specifically, rain droplets are responsible for producing a significant decrease of received signal power, often referred to as signal attenuation in engineering. This decrease depends on rainfall intensity according to the following power-law relationship ( ITU-R 2005; Olsen *et al.*, 1978):


$$\gamma =\alpha R^{\beta }\;(1)$$

where *R* is rainfall intensity in mm/h, *γ* is rain attenuation per unit path length, in units of dB/km, and *α* and *β* are coefficients dependent on CML frequency and polarisation, among others. When CMLs are used as opportunistic rainfall sensors, rain attenuation is extracted from CML raw data, and used in Equation (1) to get *R*. The raw data are received power level (RSL) and transmitted power level (TSL) across each CML. Equation (1), in practical terms, estimates signal attenuation due to rainfall by comparing RSL during rain with RSL (namely the baseline level) measured during clear-sky conditions. Moreover, before applying Equation (1), effects unrelated to rain, such as extra signal attenuation produced by wet antennas, must be filtered out. CML rainfall measurement is a spatial average across the straight path between antennas. Finally, rainfall intensity can be estimated as a (nearly) instantaneous value, an average, or an extreme value over a certain period of time, depending on the raw data collection protocol.

It is worth remarking that, thanks to the large number of CMLs in telecommunication networks, rainfall observations by CMLs can be made over country-wide areas ( Graf *et al.*, 2019; Overeem *et al.*, 2013).

TSL and RSL measurements are collected by telecom companies, usually in aggregated form and retained only for short periods, as they are essentially used for network monitoring and maintenance. What inhibits most companies from sharing these measurements are confidentiality concerns, typically regarding antenna locations, and fears of additional efforts or costs in setting up and running a data collection system, or simply lacking business models for monetizing such data. However, there are several examples in the OpenSense community of how these issues have been overcome.

### Satellite microwave links

SMLs are part of SatCom systems and are typically used for broadcasting television or for providing two-way broadband connectivity over the broad satellite footprint coverage area. Licensed frequency bands for the downlink of SatCom services are in the Ku- and Ka-bands, that is, 10-13 GHz and 18–21 GHz, respectively. When working as opportunistic rainfall sensors, SMLs rely on the same principle as CMLs, which is radio wave attenuation due to rain ( Giannetti & Reggiannini, 2021). However, there are a few important differences with respect to CMLs:

- One of the two link terminals is a GEO satellite, while the other is a ground-based user terminal ( Figure 1b). Hence, only a small fraction of the propagation path is affected by rain (a GEO satellite is located about 36,000 km above ground).
- TSL data from the satellite terminal is usually not available, hence rainfall must only be extracted from RSL or from an equivalent quantity called the signal-to-noise ratio (SNR) evaluated at the receiver site.
- RSL (or SNR) exhibit time-varying behaviour not ascribable to atmospheric effects, but due instead to the causes addressed hereafter..

In principle, links based on low/medium Earth orbit satellites could be used as opportunistic rainfall sensors, too. In this case, the space-located transmitting terminals move across the sky. However, here we consider only GEO satellite links for three reasons: 1) they are a simple case of fixed point-to-point link resembling CMLs, 2) SML reception is possible with low-cost commercial-grade equipment for direct satellite broadcasting reception, and 3) the available literature on the topic is limited to GEO satellites.

With slant propagation paths, the layered structure of the troposphere should be taken into account ( *e.g.*, including melting layer effects). A key parameter to derive rainfall estimates from an SML is rain height, that is the height above ground where all the solid precipitation melts into liquid rain, which is closely related to the 0°C isotherm height. Unwanted received signal oscillations are due to a number of factors: 1) periodic satellite housekeeping manoeuvres; 2) TSL adjustments occasionally made by the operator; and 3) apparent fluctuations of the satellite position in the sky caused by gravitational perturbations affecting the geostationary orbit. All these signal fluctuations must be filtered out by ad-hoc pre-processing of the raw data.

A signal representing clear-sky conditions, *i.e.*, an undisturbed signal level received in an ideal case of no attenuation caused by the hydrometeors during the precipitation event, is used as the reference level for rain attenuation calculation. This reference level can be evaluated analytically, *e.g.*, based upon nominal values derived from link-budget, or approximated by RSL (or SNR) before the event, or, again, computed from signal statistics of the dry days before the event ( Colli *et al.*, 2020).

An advantage of SML-based rainfall measurements is that they usually have a high temporal resolution in the order of 1 or more readings per minute. SML data can be collected by the user terminals and transferred through the terrestrial network, or they can be sent back from many user terminals through the satellite to the gateway stations and there collected. Finally, it is important to note that for each GEO broadcast satellite there is a multitude of active transponders and that tens of satellites are in visibility on the GEO arc in the sky (see Giannetti & Reggiannini, 2021, Figure 2), hence greatly expanding SML data-gathering options.

### Personal weather stations

A PWS is a privately owned weather station which can be set up by users typically comprising one, or a set of, low-cost device(s) recording meteorological variables, as shown in Figure 1. Many popular types of PWS measure rain with an unheated plastic tipping bucket rain gauge. They often have a smaller orifice opening than standard tipping bucket rain gauges ( Bárdossy *et al.*, 2021). Since these devices are not heated, their usefulness is limited to wet precipitation. The tips of the tipping bucket that correspond to a given amount of precipitation are recorded for regular or, in some cases, irregular time intervals. This information yields the total amount of precipitation for a given time interval.

The precision and accuracy of a specific measuring instrument usually follows the manufacturer's specifications. For example, for Netatmo PWS, which is one of the most common types, rain gauges can deliver observations as multiples of the volume of one tip, which, by default, is 0.101 mm, but may differ when a PWS owner has calibrated the device to a different amount. Accuracy would need to be regularly checked to ensure its preciseness ( de Vos *et al.*, 2019a). In previous studies ( de Vos *et al.*, 2017; de Vos *et al.*, 2019a), when PWS rainfall measurements were compared to co-located operational reference rain gauges, rounded measurements were found to be as accurate as operational rain gauges, provided the PWS instruments were properly installed.

PWS are an important OS source as it is possible for PWS owners to share (and visualise) observations in real-time to online platforms. Some examples are the Wundermap by Weather Underground, the Netatmo platform, the WOW-platform (or also available here), and smaller start-up companies, such as FieldSense or Weathercloud. The platforms thereby give access to large amounts of in-situ weather observations. In the case of Netatmo, the platform is provided by the company and sharing data requires very little effort which has resulted in an extremely dense international PWS network. It should be noted that while PWS observations are provided and shared by PWS owners, the data are owned by the commercial companies that maintain the platform where data are collected. For researchers and the national weather service, this means a dependency on third parties and uncertainties in the exact processing of the data.

### Other opportunistic sensors

Even though this paper focuses on three types of opportunistic sensors, it is worth noting that there are other promising technologies which could be used for the OS of precipitation. For example, recent advances in image processing have enabled quantifying rainfall intensity from recordings of surveillance cameras ( Dong *et al.*, 2017). Opportunistic sensors can also measure more environmental variables than rainfall which are, however, correlated with rain. A prominent example is the use of Global Navigation Satellite Systems (GNSS) for estimating water vapour profiles ( Guerova *et al.*, 2016). The delay of the signal propagating through the atmosphere is related to its refractive index, which, in turn, depends on water vapour content. A ground-based network of GPS receivers can provide near real-time observations suitable for assimilation into Numerical Weather Models (NWMs) and climate applications. Several studies incorporated GNSS into the monitoring of severe weather events, especially those related to precipitation ( *e.g.,* Douša *et al.*, 2016). Brenot *et al.* (2013) have presented the usefulness of GPS tropospheric gradients as preliminary signs of deep convection and, recently, GNSS time series have been used as indicators of precipitation in a climate region with a high annual precipitation amount ( Li *et al.*, 2021; Zhao *et al.*, 2018). Interest in GNSS meteorology was demonstrated in COST Action 716 “Exploitation of ground-based GPS for operational weather prediction and climate applications” (1999–2004, Elgered, 2001) and COST Action ES1206 “Advanced GNSS Tropospheric Products for Monitoring Severe Weather Events and Climate” (2013–2017, Jones *et al.*, 2020). For other OS techniques see *e.g.*, Muller *et al.* (2015), Tauro *et al.* (2018), or Zheng *et al.* (2018).

## Current practices in OS data collection

The focus of this section is on the data collection methods of opportunistic rainfall measurements and how data are stored and made available. CML and SML are part of a telecommunication network where TSL, RSL and SNR of individual links are routinely collected by network operators through specific protocols for the purposes of network monitoring and resource optimization. The above raw data are typically stored as time series. To derive rainfall information, CML and SML require additional metadata from the network ( *e.g.*, sensor position) and additional data from external sources. In contrast, PWSs provide direct rainfall measurements. Data from many PWSs are transferred to an online platform from where they are made available.

### Commercial microwave links

CML data are acquired either by a network management system (NMS) or by a tailored data acquisition (DAQ) system communicating with network elements through the simple network management protocol (SNMP). Obtaining data from NMS usually does not require the operator to set up a new application, whereas SNMP DAQ needs to be installed on a server connected to the backhaul network and operated and maintained in addition to NMS ( Chwala *et al.*, 2016). Moreover, the secure transfer of data from a backhaul network must not endanger network operations. About half of the OpenSense Action members obtain data through NMS while the remaining half use independently developed SNMP-based DAQ systems.

NMS is a platform used by the operators to monitor network operations and link quality. It includes a set of automated algorithms that adapt the network, in real time, to mitigate sudden disturbances and degradation in the transmitted signals ( Metsälä & Salmelin, 2015). NMS usually provides maxima and minima of RSL and/or TSL every 15-min. Information about the usage of Automatic Transmit Power Control (ATPC) and Adaptive Coding and Modulation (ACM) is sometimes collected by NMS and may help when estimating 15-min total losses from the RSL and TSL extremes. Some NMSs provide either RSL or TSL. If CMLs do not feature ATPC, *i.e.*, they transmit a signal at a constant TSL, RSL data alone enables rainfall retrieval, and vice versa, TSL data alone, in principle, enable rainfall retrieval if the ATPC of CMLs maintains a constant RSL. Finally, rainfall retrieval from RSL alone, in case of variable TSL, is also possible, though with some limitations, provided that some quantitative information about ATPC is available ( Roversi *et al.*, 2020).

Specially tailored DAQ systems poll CML data using the SNMP protocol, are located within the closed IP subnetwork of the CMLs and directly connect to the CMLs *via* their IP address. To poll RSL, TSL and other potential data as the outdoor unit temperature, the DAQ system sends a command called an OID (object identifier) *via* an SNMP request to the CMLs. However, the required OIDs for requesting the current RSL and TSL level are not standardised. Different hardware types, even from the same manufacturer, require different OIDs. The correct OIDs have to be identified in the MIB (management information base) file which stores the OIDs for a specific hardware or from manuals where one has to search for specific keywords such as RSL or RX. A small collection of OIDs for different hardware is given in ( Chwala *et al.*, 2016). DAQ systems poll instantaneous values of TSL and RSL at a predefined sampling frequency. Currently, systems with 1-min sampling ( Chwala *et al.*, 2016), or approximately 10 second sampling are used ( Andersson *et al.*, 2022; Fencl *et al.*, 2015).

RSL and TSL time series need to be paired with CML metadata: CML id, sublink id, site 0 and site 1 coordinates (locations of antennas), sublink frequencies and, if available, polarizations. Frequency and polarisation affect parameters *α* and *β* of attenuation-rainfall relation in Equation (1) and the coordinates uniquely define the position of a CML and its length. Other metadata such as the altitude of the terminals and antenna elevation above ground, or, again, antenna type, may also be available and could help when processing the data.

Several different systems for CML data storage and processing have, so far, been developed and operated for research purposes. A common scheme is the following: RSL and TSL collected by a network operator are provided to an external entity ( *e.g.*, a research institution or a national weather service), which is responsible for the rainfall retrieval process. Then the data are paired with metadata and converted to rain intensities using various processing methods ( Chwala & Kunstmann, 2019). In this case the CML network operator either provides access to its internal servers to the external organisation or sequentially sends the RSL and TSL data to the external servers in a push command. The data provided by DAQ systems are sent in a raw form or after a basic data quality check ( Andersson *et al.*, 2022). The data-metadata association is done in one of two ways: a) on external servers as part of the rainfall retrieval process using static metadata files updated manually on a regular basis, or b) within a data mediation system run and maintained by the network operator ( Andersson *et al.*, 2022). CML data are stored mostly in SQL databases or as text or binary files usually following the format specification of original raw data.

### Satellite microwave links

SML data collection and transfer procedures for rain monitoring is divided here into two broad categories:

A. Satellite receiver terminals (such as DTH TV broadcast receivers, *etc.*) that transmit the data through a different medium other than satellite ( *e.g.*, local Internet connection, cellular, *etc.*). As satellite bandwidth is limited, this strategy is flexible with respect to the amount of data stored and transmitted. The majority of SMLs belong to this category.
B. The satellite ground terminal operates in a two-way fashion: down-link ( *i.e.*, TV broadcast from a satellite to the terminal) and up-link ( *i.e.*, user-generated traffic plus data about the status of the device, including RSL or SNR), and no other connection apart from the satellite is available. This is the case, for instance, of interactive TV services, or satellite coverage in remote areas for Internet of Things (IoT) connectivity, FinTech and Agritech applications. The raw data are already collected for network management, *i.e.*, only the data that the terminal requires for standard operations are available, such as RSL or SNR.

Procedure B of data collection resembles the CML case where the satellite operator is the owner of the data and can provide it to the potential user. On the other hand, procedure A is somewhat independent of the operator as the received signal at ground can be transferred from the ground receiver to a central server through a terrestrial network which is not usually controlled by the satellite operator.

A typical SML receiver consists of a customary parabolic antenna ( *e.g.*, 80-cm diameter) equipped with a universal Low-Noise Block Converter (LNB) which receives the digital TV signals in DVB-S/S2 format, transmitted in Ku-band (10–13 GHz) featuring horizontal or vertical polarisation, from commercial GEO satellites ( Giannetti *et al.*, 2022). The LNB outputs a filtered/amplified and down-converted signal in L-band (1–2 GHz) which is sent *via* coaxial cable to a subsequent device for RSL or SNR measurement. Such a device can be either an *ad-hoc* device designed to make these measurements or a customised commercial receiver. In the former, it can be implemented with a low-power IoT electronic board managing data acquisition and transmission functionalities ( Colli *et al.*, 2019). In the latter, it consists of a commercial device, suitably modified to make its RSL or SNR measurements available and interfaced with an external router for data logging *via* a terrestrial mobile network ( Giannetti *et al.*, 2022).

A highly appealing solution for implementing an SML consists of using a new-generation Eutelsat IoT FIRST (formerly named SmartLNB) commercial receiver ( Giannetti *et al.*, 2019). The satellite infrastructure is courtesy of a EUROBIS platform, a bidirectional, satellite-based interactive system owned and operated by Eutelsat. EUROBIS, operating in Ku band and linear polarisation using a Eutelsat 10A satellite in a 10° E orbital position, provides IP-based, transparent communication between the satellite receivers and the Internet. SNR measurements are collected every 30 seconds by the SMLs equipped with Iot FIRST receivers and, subsequently, sent to a fusion centre via the EUROBIS platform through the satellite return link. From there, the user can download the SML data as text files.

RSL (or SNR) time series need to be paired with SML metadata: SML id, site 0 coordinates ( *i.e.*, the location of the ground antenna), antenna elevation above ground and antenna altitude above sea level, site 1 ( *i.e.*, GEO satellite) latitude and rain height, frequency and polarisation. Other metadata such as digital modulation and error-correcting code format could be helpful as well.

### Personal weather stations

A PWS most commonly features a tipping bucket rain gauge which directly measures rain volume by recording bucket tips within some time interval. These outdoor observations are usually transmitted wirelessly to an indoor module over a distance of up to 100 m. The indoor module then transfers the observations to an online platform at a particular set time interval. The rainfall observation always describes the number of tips in the past interval which leads to a delay between the actual time of observation and time of transmission. Another delay is due to data processing and handling by the platform before the output data are available on the server.

In many cases, historical PWS data can be accessed online via an API or purchased from the provider ( *e.g.*, as was done for the Amsterdam PWS data processed by de Vos (2019b) in the OpenSense sandbox). In addition, EUMETNET has provided two Europe-wide rainfall data sets from Netamo and WOW platforms for the year 2020 (available here). For operational purposes, Netatmo offers services to obtain (near) real-time PWS data if a corresponding agreement with the company is signed.

The most common format of PWS data is text files (CSV, JSON) with limited metadata information, typically including station IDs, coordinates of the site and, occasionally, the altitude of the site. Temporal resolution of data can differ. For example, from the Netatmo API rainfall observations can be obtained as raw data with irregular timestamps (~5 minutes), processed datasets with homogeneous fixed 5 minutes, hourly or daily intervals. True clarity on how the data are aggregated and processed is lacking.

## Proposed data and metadata standards

Building from the experience of the OpenSense community, this section provides recommendations for defining common data and metadata standards and formats for CML, PWS, and SML to enable effective rainfall monitoring by OS. We distinguish among required, recommended, and optional variables. The required variables represent the minimal information needed to make use of OS data for rainfall monitoring. Recommended variables are not essential to gather rainfall observation but they improve its accuracy and reliability. Finally, optional variables are currently not used by common processing methods, but they are potentially relevant for improving rainfall monitoring in the future or for monitoring other environmental variables. Sometimes, variables not inherent to the sensor or system of sensors is necessary to derive rainfall information ( *e.g.*, rain height by SMLs). We call such variables external and recommend including them in the sensor’s dataset.

### Commercial microwave links

The CML dataset shall include two main types of data:

1. The metadata table contains a set of parameters characterising the link in terms of its position and other features, such as frequency, polarisation or hardware.
2. The raw data, *i.e.*, the set of dynamic measurements that the CML records such as RSL or TSL.

Raw data are time series, hence they are associated with a timestamp. The metadata table does not have a timestamp but is not static. It can be updated by the cellular operator following changes in the set of operational links. Raw data and metadata are, strictly, all the relevant information for OS of rainfall that can be gathered from the system controlling the network of opportunistic sensors.

***Required data***

The minimal set of data shall include the following records:

- **(metadata) Location**: The transmitter and receiver coordinates (latitude and longitude) in decimal degrees (°). The location is crucial for determining the path length of the sensor (in km) and the geographical position.
- **(metadata) Link identifier**: A string uniquely identifying a link in a network. It associates time series in the raw data with link features in the metadata.
- ( **metadata**) **Sublink identifier**: a string that identifies the sublink channel.
- **(metadata) Frequency**: The carrier frequency (MHz) is necessary to relate through Equation (1) signal attenuation across the CML path with rainfall intensity.
- **(raw data)** **Timestamp** (UTC time)
- ( **raw data) RSL and/or TSL**: The RSL (in dBm) is the power measurement recorded at the receiver end of the link. The variations in the RSL during a rain event are correlated with the corresponding changes in rainfall intensity.

Even though it is recommended to store both TSL and RSL, in the following two cases just one of these variables used alone enables rainfall retrieval: i) when TSL is maintained as a constant only RSL records are required, ii) when RSL is maintained as a constant, only TSL records alone are required. The latter may not work with moderate to heavy rainfall due to the limited ATPC range usually available. Hence, it is recommended to provide information about an upper limit to which TSL is increased by ATPC.

***Recommended data***

To gather more accurate rainfall estimates from CMLs, it is also recommended to collect the following data types:

- **(metadata) Signal polarisation**. It is usually either vertical or horizontal.
- **(metadata) Antenna altitude above mean sea level** of each link terminal (m).

***Optional data***

In addition to RSL and TSL, several other raw data types can be made available by the NMS, such as link failure, ATPC, ACM, sensor temperature, and MIMO information ( Andersson *et al.*, 2022; Roversi *et al.*, 2020). Other potentially useful metadata are hardware characteristics such as antenna dimensions, device manufacturer and model, date of installation, and information about the presence, type and state of protective antenna radomes (whose wetting increases the measured attenuation during rain).

### Satellite microwave links

Even though both CML and SML are microwave links, there are important differences between a CML and an SML when it comes to the OS of rainfall. These differences were highlighted in the “opportunistic rainfall sensors” and “current practices in OS data collection” sections and reflect upon different sets of metadata and raw data.

***Required data***

- **(metadata) Location of the ground receiver and satellite position**: latitude and longitude (in decimal degrees) of the ground receiver, and satellite longitude position (assuming a geostationary orbit). These data determine the elevation angle of the link which is used to determine the length of the slant path through rain.
- **(metadata) Altitude of the ground receiver** (m) above mean sea level.
- **(external data) Rain height** (m): determines the length of the slant path through rain.
- **(metadata) Link identifier**: a string that uniquely identifies a link in a network. It associates time series in raw data with link features in the metadata.
- ( **metadata**) **Sublink identifier**: a string that identifies the sublink channel.
- **(metadata) Frequency** (in MHz)
- **(metadata) Polarisation** (linear V/H in C-, Ku- and Ka-band; circular L/R in C- and Ka-band) of the downlink carrier used for rain attenuation measurements.
- **(raw data)** **Timestamp** (UTC time)
- **(raw data) RSL (dBm) or SNR (dB)**: alternative measurements of the signal strength recorded by the ground receiver.

The rain height information is not an SML-based output. It is, however, in the list of required data as it is mandatory to calculate the length of the propagation path across the liquid precipitation. The rain height can be derived in several ways: i) from the knowledge of the 0°C isotherm height, minus the thickness of the melting layer; ii) from a short-term forecast based on numerical weather prediction models; iii) from atmospheric profiling; iv) it can be set as a fixed value based on daily or yearly averages *e.g.*, as specified in ITU-R recommendations ( ITU-R, 2013), taken at the location of the ground receiver.

***Recommended data***

- **(metadata) MODCOD** (digital modulation and error-correcting code) format. The combination of modulation and coding determines receiver sensitivity, *i.e.*, the required minimum SNR for proper operation at target error rate. This value expresses the robustness of the receiver to rain fades. The lower the required SNR, the wider the operating range of the receiver as a rain intensity measurement instrument.
- **(metadata) Minimum SNR ratio** (in dB). The required minimum value for SNR which allows proper operation at a target error rate.

### Personal weather stations

As PWS are owned and managed at a non-professional level, the data gathered from different stations can largely differ in type, size and quality. Hence, the data that is required for use is limited. Depending on the data source there may be more data relevant for the user which can be added optionally in the data format. The best practice is to follow the recommendations of the Guide to Meteorological Instruments and Methods of Observation edited by the World Meteorological Organization, (WMO-No. 8), Guide to Instruments and Methods of Observation (2021).

***Required data***

- **(metadata)** **Location**: PWS horizontal coordinates (latitude and longitude) in decimal degrees (°).
- **(metadata)** **PWS identifier**: an alphanumeric tag that uniquely identifies the station. It associates time series in raw data with sensor features in the metadata.
- **(raw data) Timestamp** (UTC time)
- **(raw data) Rainfall tips/rainfall accumulation**.

The minimal required metadata for a PWS are its coordinates and a unique identifier. Guidelines for the setup and placement of a PWS are provided by the manufacturers but these guidelines may differ from WMO standards and in general, there is no control on how individual stations are set up. As many PWS are installed in built-up areas (cities), it is likely that PWS placement does not follow WMO standards. In the case of Netatmo, an automatic site location and elevation value are generated by the platform when first installing the PWS. If the PWS owner does not manually change this metadata, it may lead to incorrect location attribution. Furthermore, as the elevation metadata can be automatically attributed, it may mean different things, *e.g.,* station height relative to ground, mean sea level, altitude of the site, therefore this value should not be taken at face value.

There may be additional meteorological variables that are being simultaneously measured, *e.g.*, air temperature, wind speed and direction, relative humidity, air pressure, which can be used for the quality control of rainfall data. It should be noted that if the PWS consists of multiple modules, other meteorological variables are measured by a sensor that may be tens of metres away from the rainfall sensor. Regardless, only one set of location metadata will be given for such a PWS.

For PWS, information on the exact siting of the device can help interpret the data, but it is not a requirement to make use of the data. It is recommended to include any available metadata, with the station location being the only required parameter. Missing data is quite common with the use of PWSs. Thus, it is important to have access to the data in the highest temporal resolution and if observations have been aggregated to larger time steps, to provide information on the calculation choices regarding the number of samples within the time window that may be missed while still generating a rainfall sum over the interval.

## Proposed data formats

Most of the OS rainfall data in the literature and the ones available on public online repositories are in either of the two following formats:

- netCDF
- CSV

netCDF is a format developed for creating, accessing, and sharing array-oriented scientific data ( UniData, 2021). It is commonly used in meteorology, climatology and hydrology. netCDFs are self-describing and machine-independent, supporting easy data exchange between researchers with different systems and programming languages. In contrast, CSV, being a list of characters separated by commas, is a human-centred format that can be easily understood, but custom data handlers have to be switched in front of the OS software.

NetCDF was chosen as a common data format because it is self-describing, *i.e.* contains all the data and metadata in one file, it is widely used by the climate- and weather-forecast community, and can be interpreted by a wide range of standardised software.

### netCDF Format

The netCDF OS data format conventions are maintained on GitHub and the reader should refer to this repository for the complete up-to-date file structure specifications of the three discussed sensors and for the most recent version of the conventions. This repository also contains examples of files and Python scripts to produce netCDF files according to OS data format conventions. The following text describes the main components of the recommended netCDF structure.

The netCDF file comprises the following components: global attributes, dimensions, coordinate variables, auxiliary coordinate variables, data variables, and external data variables.

- **Global attributes** contain ancillary information describing stored data and should follow the standard structure which is in line with CF conventions ( Hassell *et al.*, 2017). For detailed information one should refer to Table 1 on GitHub ( netCDF_global_attributes.adoc).
- **Dimensions** represent the shape of N-dimensional data variables. Their size is defined either as unlimited (typically by time dimension) or it is an arbitrary positive integer.
- **Coordinate** variables are 1-D variables with the same name as the dimension. They contain the coordinate values of a dimension.
- **Auxiliary coordinate variables** contain coordinate data, but are not coordinate variables in the sense defined above. They do not share a name with dimensions and can be multidimensional. Coordinate variables mostly contain metadata about the sensor, *e.g.*, geographical coordinates, or some sensor characteristics such as frequency for CMLs or type of sensor for PWS.
- **Data variables** are N-dimensional arrays that contain the observations of the sensors ( *e.g.* the RSL time series in the case of CMLs)
- **External data variables** contain data from external sources, which are required (or recommended to be used) for data processing

Global attributes common to all three sensors are shown in Table 2. Table 3 shows specifications for CMLs, Table 4 for SMLs, and Table 5 for PWSs. The tables correspond to the OS data format conventions v0.1 maintained at GitHub The column named Attributes includes properties common to all variables as measurement units (when pertinent) and a string descriptor ( long_name) as well as properties specific to certain variable types. For a data variable, the coordinate attribute is a string containing the auxiliary coordinate variables that belong to the data variable ( *e.g.* the rainfall amount coordinate attribute by PWS would be longitude latitude height_above_ground_level environmental_class hardware). The missing_value attribute is the conventional value of missing data. It is not set here, but the user should define it. The recommendation is to use, *e.g.*, “NA” or “na”. Coordinate and data variables are classified into required, recommended and optional (fourth column in Table 2, Table 3, and Table 4), as discussed in the section “Proposed data and metastandaards”.

**Table 2.** global attributes of netCDF files.

Attribute name	Specification	Requisite
title	brief description of what is inside the dataset	Recommended
file_author(s)	who produced the data and contact	Recommended
institution	where the dataset was produced	Recommended
date	when the dataset was created	Recommended
source	the method of production of the original data. If it is model-generated, the source should include the model and its version. If data are gathered from observations, the source record should characterise it ( *e.g.*, "surface observation" or "radiosonde")	Recommended
version	version of the dataset (version number or name)	Recommended
history	any modification of the data; the timestamp should be provided for each modification	Recommended
naming convention	the conventional name for OS data is OpenSense-X	Recommended
licence	under which licence the data are available	Recommended
reference	data source or doi, if available	Optional
comment	diverse information about the dataset ( *e.g.*, precision of coordinates, the time period of the campaign)	Optional

**Table 3.** CML netCDF specification.

	Type	Attributes	Requisite	Comments
**Dimensions**				
time				Unlimited size, enforce UTC seconds since 1970-01-01
cml_id				Minim. length is 1
sublink_id				Minim. length is 1
**Coordinate variables (dimension)**				
time (time)	int/float/double	units = "seconds since 1970-01-01 00:00:00 UTC", long_name = “time_utc”, missing_value	Required	
cml_id (cml_id)	string	long_name = “commercial_microwave_link_identifier”	Required	cml_id has to be unique across the network
sublink_id (sublink_id)	string	long_name = “sublink_identifier”	Required	sublink_id does not have to be unique across the network (but unique within each CML)
**Auxiliary coordinate variables (dimension)**				
site_0_lat (cml_id)	float/double	units = degrees_in_WGS84_projection, long_name = “site_0_latitude”	Required	
site_0_lon (cml_id)	float/double	units = degrees_in_WGS84_projection, long_name = “site_0_longitude”	Required	
site_0_elev (cml_id)	float/double	units = metres_above_sea, long_name = “ground_elevation_above_sea_level_at_site_0”	Optional	
site_0_alt (cml_id)	float/double	units = metres_above_sea, long_name = “antenna_altitude_above_sea_level_at_site_0”	Recommended	
site_1_lat (cml_id)	float/double	units = degrees_in_WGS84_projection, long_name = “site_1_latitude”	Required	
site_1_lon (cml_id)	float/double	units = degrees_in_WGS84_projection, long_name = “site_1_longitude”	Required	
site_1_elev (cml_id)	float/double	units = metres_above_sea, long_name = “ground_elevation_above_sea_level_at_site_1”	Optional	
site_1_alt (cml_id)	float/double	units = metres_above_sea, long_name = ”antenna_altitude_above_sea_level_at_site_1”	Recommended	
length (cml_id)	float/double	units = m, long_name = “distance_between_pair_of_antennas”	Optional	
frequency (cml_id, sublink_id)	float/double	units = MHz, long_name = “sublink_frequency”	Required	
polarisation (cml_id, sublink_id)	string	units = no units, long_name = “sublink_polarization”	Recommended	When string then ‘vertical’ or ‘horizontal’
**Data variables (dimension)**				
*Specifications for instantaneous sampling (SNMP DAQ)*				
tsl (cml_id, sublink_id, time)	float/double	units = dBm, coordinates = string_with_auxiliary_coordinate_variable_names, long_name = "transmitted_signal_level", sampling = 'instantaneous', missing_value	Required *	
rsl (cml_id, sublink_id, time)	float/double	units = dBm, coordinates = string_with_auxiliary_coordinate_variable_names, long_name = "received_signal_level", sampling = 'instantaneous', missing_value	Required *	
Specification for min/max sampling (NMS DAQ) **				
tsl_max (cml_id, sublink_id, time)	float/double	units = dBm, coordinates = string_with_auxiliary_coordinate_variable_names, long_name = "maximum_transmitted_signal_level_over_time_window", sampling = 'aggregated', missing_value	Required *	
tsl_min (cml_id,sublink_id, time)	float/double	units = dBm, coordinates = string_with_auxiliary_coordinate_variable_names, long_name = "minimum_transmitted_signal_level_over_time_window", sampling = 'aggregated', missing_value	Required *	
tsl_avg (cml_id, sublink_id, time)	float/double	units = dBm, coordinates = string_with_auxiliary_coordinate_variable_names, long_name = "averaged_transmitted_signal_level_over_time_window", sampling = 'aggregated', missing_value	Optional	
rsl_max (cml_id, sublink_id, time)	float/double	units = dBm, coordinates = string_with_auxiliary_coordinate_variable_names, long_name = "maximum_received_signal_level_over_time_window", sampling = 'aggregated', missing_value	Required *	
rsl_min (cml_id, sublink_id, time)	float/double	units = dBm, coordinates = string with metadata variable_names, long_name = "minimum_received_signal_level_over_time_window", sampling = 'aggregated', missing_value	Required *	
rsl_avg (cml_id, sublink_id, time)	float/double	units = dBm, coordinates = string_with_auxiliary_coordinate_variable_names, long_name = "averaged_received_signal_level_over_time_window", sampling = 'aggregated', missing_value	Optional	
temperature_0 *** (cml_id, time)	float/double	units = degrees_of_celsius, coordinates = string_with_auxiliary_coordinate_variable_names, long_name = “sensor_temperature_at_site_0”	Optional	
temperature_1 *** (cml_id, time)	float/double	units = degrees_of_celsius, coordinates = string_with_auxiliary_coordinate_variable_names, long_name = “sensor_temperature_at_site_1”	Optional	

* It is recommended to store both TSL and RSL, however, when TSL or RSL is maintained as constant, only the variable which is changing is required. ** if other aggregation satistics is used ( *e.g.* mean, median), create a variable rsl_nameOfAggregationStatistics/tsl_nameOfAggregationStatistics and specify details in the global attribute 'comment' *** Names of variables related to site conditions, such as temperature, should be distinguished by suffixes 0 and 1.

**Table 4.** SML netCDF specification.

	Type	Attributes	Requisite	Comments
**Dimensions**				
time				Unlimited size, enforce UTC seconds since 1970-01-01
sml_id				Minimum length is 1
sublink_id				Minimum length is 1
**Coordinate variables (dimension)**				
time (time)	int/float/double	units = seconds since 1970-01-01 00:00:00 UTC, long_name = “time_utc”, missing_value	Required	
sml_id (sml_id)	string	long_name = “satellite_microwave_link_identifier”	Required	sml_id has to be unique across the link. It includes: receiver_id, satellite_id, trasponder_id
sublink_id (sublink_id)	string	long_name = “sublink_identifier”,	Required	sublink_id does not have to be unique across the network (but unique within each SML)
**Auxiliary coordinate variables (dimension)**				
site_0_lat (sml_id)	float/double	units = degrees_in_WGS84_projection, long_name = “site_0_latitude”	Required	
site_0_lon (sml_id)	float/double	units = degrees_in_WGS84_projection, long_name = “site_0_longitude”	Required	
site_0_alt (sml_id)	float/double	units = degrees_in_WGS84_projection, long_name = ”antenna_altitude_above_sea_level_at_site_0”	Required	
site_1_lat (sml_id)	float/double	units = degrees_in_WGS84_projection, long_name = “site_0_latitude”	Optional	0 degrees
site_1_lon (sml_id)	float/double	units = degrees_in_WGS84_projection, long_name = “site_1_longitude”	Required	
site_1_alt (sml_id)	float/double	units = metres_above_sea, long_name = “ground_elevation_above_sea_level_at_site_1”	Optional	36,000 km
frequency (sml_id, sublink_id)	float/double	units = MHz, long_name = “sublink_frequency”	Required	
polarisation (sml_id, sublink_id)	string	units = no units, long_name = “sublink_polarization”	Required	When string then ‘vertical’ or ‘horizontal’
**Data variables (dimension)**				
snr (sml_id, time)	float/double	units = dB, coordinates = string_with_auxiliary_coordinate_variable_names, long_name = "signal_to_noise_ratio", missing_value	Required *	
rsl (sml_id, time)	float/double	units = dBm, coordinates = string_with_auxiliary_coordinate_variable_names, long_name = "received_signal_level", missing_value	Required *	
modcod (sml_id, time)	string	long_name = “modcod_format”	Optional	
**External data variables (dimension)**				
rain_height (time, sml_id)	float/double	units = m, long_name = “length_of_the_slant_path_through_the_rain”	Required	

*It is required to store either snr or rsl.

**Table 5.** PWS netCDF specification.

	Type	Attributes	Requisite	Comments
**Dimensions**				
time				Unlimited size, enforce UTC seconds since 1970-01-01
id				
**Coordinate variables (dimension)**				
time (time)	int/float/double	units = seconds since 1970-01-01 00:00:00 UTC, long_name = “time_utc”, missing_value	Required	Timestamp refers to the end of observation interval
id (id)	string	long_name = “personal_weather_station_identifier”	Required	id has to be unique across the network
**Auxiliary coordinate variables (dimension)**				
latitude (id)	float/double	units = degrees in WGS84 projection	Required	
longitude (id)	float/double	units = degrees in WGS84 projection	Required	
elevation (id)	float/double	units = metres_above_sea	Recommended	
Height_above ground_level (id)	float/double	units = metres	Recommended	
Environmental_class (id)	integer	no unit	Recommended	
hardware (id)	string	long_name = “manufactuer_and_model_type”	Optional	*e.g.* manufacturer, station type, sensor types
**Data variables (dimension)**				
rainfall_amount (id, time)	float/double	long_name = “rainfall_amount_per_time_unit”, units = mm, coordinates = string_with_auxiliary_coordinate_variable_names	Required	
**Optional data (dimension)**				
temperature (id, time)	float/double	units = degrees_celsius	Optional	
relative_humidity (id, time)	float/double	units = %	Optional	
wind_velocity (id, time)	float/double	units = ms-1	Optional	
wind_direction (id, time)	float/double	units = degrees	Optional	
air_pressure (id, time)	float/double	units = hPa	Optional	

Creating one file with multiple sensors stored along the sensor’s id dimension is recommended only if all sensor data have the same timestamps. In case the timestamps differ, it is recommended to locate each sensor in one netCDF group (supported by netCDF-4) or to create one file for each sensor. The length of the sensor's id dimension is then equal to 1.

The netCDF file for CML data is split over three dimensions: time period ( time), CML identifier ( cml_id), and sublink identifier/s ( sublink_id). The conventions slightly differ for data obtained by instantaneous and min-max sampling of RSL and TSL. In the first case, data are stored in rsl and tsl variables and instantaneous sampling is indicated in the sampling attribute of the variables, in the second case, rsl_min, rsl_max, tsl_min, and tsl_max variables are introduced instead of rsl and tsl and the fact that the values represent statistics over whole observation intervals is indicated in the sampling attribute. If some other statistics than min/max is used to describe observation intervals, a user can define a new variable name, *e.g.* rsl_avg, or rsl_median and describe the details about this variable in the global attribute comment.

The netCDF file for SML data is split over the following dimensions: time period ( time), ground receiver identifier ( receiver_id), satellite identifier ( satellite_id), transponder identifier ( transponder_id), and sublink identifier ( sublink_id).

The netCDF file for PWS data has two dimensions: a timestamp ( time) and a PWS identifier ( id). Creating one file with multiple PWSs stored along the id dimension is recommended only if PWS data have the same timestamps. For PWSs that start later in the period specified by the time dimension, the first intervals are filled with NA-values. If PWSs timestamps differ, it is recommended to store each PWS in a netCDF group (supported by netCDF-4) or to create a file for each PWS. If the optional data consisting of additional meteorological variables have a different timestamp, it is recommended to interpolate these variables at the timestamps of the rainfall data to have a structured, fixed time series.

### CSV Format

The proposal is to follow naming conventions and units defined in netCDF but not to enforce any other requirements on the structure. A header of the table should be present. As in the netCDF files, the format of the missing value names is not set, but it should be defined. The recommendation is to use *e.g.*, “NA” or “na”. It is also highly recommended to include a Readme file with a description of general information concerning the data, as covered in the global attributes of netCDF files ( *i.e.*, title, author/s, institution, date, *etc.*).

***Commercial microwave links***

The proposal is to follow the naming conventions defined for netCDF as much as possible (see section “netCDF format”), but not to enforce any other requirements on the structure. The character of the CSV files does not allow for any direct inclusion of metadata of individual sensors. Therefore, a metadata table is needed for the storage of information covered in the data coordinates of the netCDF files ( *e.g.*, coordinates of sites, CML length, frequency, polarisation, *etc.*), preferably with the same naming convention. Another table should store the time series of the CML variable observations.

***Satellite microwave links***

The recommendations concerning naming and metadata are similar to those for CML. As for storing the data collected by a network of SML sensors, we recommend following the approach described in F. Giannetti *et al.* (2019) where each sensor produces its own CSV file.

***Personal weather stations***

If PWS data are provided in CSV form, the data should be divided over two tables with the first consisting of the data where each column represents the time series of a single PWS. The first column of the table is to indicate the timestamps of the end of the interval. This CSV format anticipates some pre-processing of rainfall observations to fixed time intervals. In the case of multiple observations within a time interval, the rainfall amounts are added. If no observation is reported in the fixed time interval, the value in the table for that timestamp becomes NA. In another CSV table, metadata are provided for each PWS. Each row corresponds with the PWS id, provided in the first column, followed by columns of the respective longitude, latitude, elevation (if possible) and any other metadata related to that PWS that may be available, including information about the originator of the data ( *e.g.*, name, contact number, *etc.*).

The CSV format does not allow for overarching metadata as the netCDF format does. A good example of a structure of PWS data and metadata in the CSV format is the Amsterdam PWS dataset by de Vos (2019b). This dataset is also available in the OpenSense SANDBOX.

## Conclusions and outlook on OS data application

This paper presents the current practices of collecting and storing precipitation OS data and corresponding metadata. For the first time, we introduce common guidelines defining i) requirements on data and metadata collected from CMLs, SMLs, and PWSs, ii) conventions for naming collected variables and different parameters of metadata, and iii) specifications on data format used for storing the data and metadata in files. These guidelines will enable storing OS datasets in a standardised form, thus easing their processing, sharing, and integration. Common naming conventions will facilitate communication of OS research and its applications. Specifications defining recommended and required data and metadata will improve the reliability of OS datasets and their quality control. The OS format conventions are maintained at GitHub repository of OpenSense Action and the reader is referred there for the most recent version.

The definition of common netCDF specifications represents an important step towards automated processing of OS raw data and community development of joint OS software packages. netCDF is a self-describing machine-readable binary format enabling the efficient handling of potentially large datasets at different platforms. The OpenSense community is currently collaborating on collecting OS software packages which have been developed by individual researchers and on harmonising their usage within a shared SANDBOX environment. A common definition of data formats is crucial for the future interoperability of developed software and automated processing of OS data collected. We also recommended how to store data and metadata in human-readable CSV files as it is a format which most users can read and is a widely used data format in open science. With the definition of CSV files, we do not aim for a unique specification of the data structure, but to give general recommendations which will later ease the conversion of these files to uniquely defined netCDF files.

These guidelines will simplify the uptake of opportunistic sensors as a relevant source of rainfall observations which can complement existing standard monitoring systems and improve our understanding of Earth’s water cycle.

## Ethics and consent

Ethical approval and consent were not required.
