# Supplementary material for: Data formats and standards for opportunistic rainfall sensors
Source: Open Res Eur. 2024 Feb 13;3:169. Originally published 2023 Oct 10. [Version 2] doi: 10.12688/openreseurope.16068.2 (PMC10884596; doi:10.12688/openreseurope.16068.2)
Supplement: Peer review history

# Reviewer report: Reviewer response for version 2

**DOI:** 10.21956/openreseurope.18616.r38145

**Published:** 2024-02-22

**Authors:** Swastika Chakraborty

**Affiliations:** 1 Electronics and Communication Engineering Department, Narula Institute of Technology, Kolkata, West Bengal, India

The revisions are appropriate. I have read the article. They are sufficient for me.

Where applicable, are recommendations and next steps explained clearly for others to follow? (Please consider whether others in the research community would be able to implement guidelines or recommendations and/or constructively engage in the debate)

Yes

Does the article adequately reference differing views and opinions?

Yes

Are all factual statements correct, and are statements and arguments made adequately supported by citations?

Yes

Is the rationale for the Open Letter provided in sufficient detail? (Please consider whether existing challenges in the field are outlined clearly and whether the purpose of the letter is explained)

Yes

Is the Open Letter written in accessible language? (Please consider whether all subject-specific terms, concepts and abbreviations are explained)

Yes

Reviewer Expertise:

Satellite Communication, Climate Studies

I confirm that I have read this submission and believe that I have an appropriate level of expertise to confirm that it is of an acceptable scientific standard.

# Reviewer report: Reviewer response for version 1

**DOI:** 10.21956/openreseurope.17348.r36009

**Published:** 2023-11-16

**Authors:** Swastika Chakraborty

**Affiliations:** 1 Electronics and Communication Engineering Department, Narula Institute of Technology, Kolkata, West Bengal, India

The article addresses a very important issue about rainfall ground truth information collection  through opportunistic sensor and dissemination of it. The common data format as proposed here (.csv and NETCDF) is necessary. According to me for the maximum coverage of spatio-temporal information the collection process should not be limited to continental boundary, neither it is limited to government organization. To my knowledge CML is maintained mostly by private organizations for their purpose. They are not for preserving continuous data. Therefore, some policy is needed to encourage private operator for the purpose to participate to the network. CCTV is an important, economic device, only it needs a proper policy guideline to use the existing network across the globe.

 An international controlling forum like ITU is required to be formed to avoid redundancy of data collection and to make aware the user about the data usage policy. The three types of data that described here i.e. required data, redundant data and optional data are enough for the purpose of rainfall research. However, collection of the data of instruments like RADAR of different type, Laser Precipitation Monitor, Disdrometer, Radiometer may add value for the purpose.

 Data storage policy and data charging policy for procurement needs to be proposed.

 Major Comments:

1. Insert separate paragraphs looking at user’s requirement, i.e. what are the steps to be performed to use the data, how the data to be acknowledged, how the charging process to be satisfied.
2. It is better to include a flow diagram for the data user.
3. More types of sensors as discussed are to be included.
4. Discuss significance of data resolution on the application in a paragraph

Minor Comments:

 Include section and sub section numbering.

Where applicable, are recommendations and next steps explained clearly for others to follow? (Please consider whether others in the research community would be able to implement guidelines or recommendations and/or constructively engage in the debate)

Yes

Does the article adequately reference differing views and opinions?

Yes

Are all factual statements correct, and are statements and arguments made adequately supported by citations?

Yes

Is the rationale for the Open Letter provided in sufficient detail? (Please consider whether existing challenges in the field are outlined clearly and whether the purpose of the letter is explained)

Yes

Is the Open Letter written in accessible language? (Please consider whether all subject-specific terms, concepts and abbreviations are explained)

Yes

Reviewer Expertise:

Satellite Communication, Climate Studies

I confirm that I have read this submission and believe that I have an appropriate level of expertise to confirm that it is of an acceptable scientific standard, however I have significant reservations, as outlined above.

# Response

**Published:** 2024-01-29

**Authors:** Martin Fencl

**Affiliations:** Department of Hydrology and Hydraulics, Czech Technical Unversity in Prague, Prague 6, Czech Republic

Response to the comments *We thank both reviewers and one reader, Pinhas Alpert, for their valuable comments. We respond to the comments at respective places in the text below. Both reviews are raising questions related to availability of opportunistic sensing (OS) data. While OS data availability is intentionally not at the center of our paper, which focuses on OS data standards and formats, we understand that it is a crucial aspect for a reader (potential end user) who does not yet have access to OS data. Thus, we address the issue of OS data availability in more detail in the introducing sections of the revised version of the manuscript.* **Reviewer Report II** **Approved with Reservations** Swastika Chakraborty, Electronics and Communication Engineering Department, Narula Institute of Technology, Kolkata, West Bengal, India   The article addresses a very important issue about rainfall ground truth information collection  through opportunistic sensor and dissemination of it. The common data format as proposed here (.csv and NETCDF) is necessary. According to me for the maximum coverage of spatio-temporal information the collection process should not be limited to continental boundary, neither it is limited to government organization. To my knowledge CML is maintained mostly by private organizations for their purpose. They are not for preserving continuous data. Therefore, some policy is needed to encourage private operator for the purpose to participate to the network. CCTV is an important, economic device, only it needs a proper policy guideline to use the existing network across the globe. 

 An international controlling forum like ITU is required to be formed to avoid redundancy of data collection and to make aware the user about the data usage policy. The three types of data that described here i.e. required data, redundant data and optional data are enough for the purpose of rainfall research. However, collection of the data of instruments like RADAR of different type, Laser Precipitation Monitor, Disdrometer, Radiometer may add value for the purpose.

 Data storage policy and data charging policy for procurement needs to be proposed.

*We thank the reviewer for all the comments. While we acknowledge the importance of new topics raised by the reviewer, we intend to have our paper focused primarily on new data format conventions for three selected opportunistic sensors. We also review data collection practices, nevertheless again with the focus on three selected sensors and with the focus on the technical aspects of the data acquisition.* *The paper arises from the experience of OpenSense network, which tackles the challenge of OS data availability and accessibility at different levels. OpenSense network involves now about eight European national meteorological services and their organization EUMETNET, many OS researchers, but also several providers of OS data. Common data format conventions, discussed in this paper, are considered by the members of the network as a major step towards improving OS data interoperability and thus represents a significant contribution towards a global uptake of OS data and its integration into standard weather observation networks.*   Major Comments:

1. Insert separate paragraphs looking at user’s requirement, i.e. what are the steps to be performed to use the data, how the data to be acknowledged, how the charging process to be satisfied.

 
*We partly address the user perspective in a new data availability section. In here, we describe how one can obtain historical open access data from CMLs and discuss also the possibility to buy data from data-collection platforms operated by PWS manufacturers. In the case of CMLs, negotiations between several met offices and mobile network operators to get CML data operationally are now in process, nevertheless, details are confidential. Regarding SMLs, there are few private companies collecting this data on their own and providing services based on this data. Business models for monetizing OS data including charging strategies are not in the hands of OpenSense* *members, but in the hands of data owners and we do not have information to reliably comment on it. Moreover, we think this issue would be beyond the scope of this paper. On the other hand, we are convinced, that suggested data formats might ease upscaling of OS data business, as they improve data interoperability and make easier for end users to use software tools being currently developed within OpenSense* *community. In the revised manuscript, this is now stressed more in the concluding section.* *Regarding processing of OS data (‘the steps to be performed to use the data’), a reader is referred to literature provided in the introduction.*  

1. It is better to include a flow diagram for the data user.

 
*As explained above, the authors of this manuscript are not in the position to propose a technical solution, how data is/will be delivered to end users.*  

1. More types of sensors as discussed are to be included.

 
*The OpenSense community can act as a reference group for usage of data from three OS sensors discussed in this paper (CMLs, SMLs, and PWSs). Thus, we do not plan to include for now other sensors into our standards. That said, we are aware that that the field of OS is dynamically evolving and other sensors might become commonly used in future. Data format conventions maintained on* *GitHub*** *presented in this paper will hopefully contribute also to the other future OS data standardization efforts.* *For traditional sensors, mentioned in the general comment, CF conventions provide widely accepted data format standards.*  

1. Discuss significance of data resolution on the application in a paragraph

*Data resolution is mentioned in the new paragraph estimating number of sensors worldwide, in addition common data resolution of different sensors is provided in Table 1. To keep the conclusion focused on the subject of this paper, i.e. new data format conventions, we prefer to keep information about typical temporal resolution of discussed OS sensors in the introducing section.*   Minor Comments:

 Include section and sub section numbering. *Section numbers have been removed by the handling editor as it does not comply with ORE style.*

# Reviewer report: Reviewer response for version 1

**DOI:** 10.21956/openreseurope.17348.r35561

**Published:** 2023-10-23

**Authors:** Cristian Lussana

**Affiliations:** 1 The Norwegian Meteorological Institute, Oslo, Norway

The authors represent the OpenSense community, which comprises researchers and professionals primarily engaged in the public sector, including governmental institutions and universities, among others. In their paper, the authors share valuable insights into the use of opportunistic sensors for precipitation measurement. I appreciate that the authors provide concise summaries of the three data sources discussed in the paper: CML, SML, and PWS. The paper's central focus lies in recommendations regarding the typical file structure necessary for data storage. I concur with the authors' fundamental idea that adopting a common format can facilitate data sharing among OpenSense members and potentially extend to the broader scientific community. The suggested formats, both netCDF and CSV file formats, seem reasonable and well-suited for this purpose.

 However, a limitation of this paper is that it does not delve into the interaction between private companies providing the data, a scenario that frequently occurs, and the public institutions that utilize them. As a reader, I am left wondering if the proposed recommendations hold value for data providers. Do they perceive the benefits of organizing data according to the proposed structure? Moreover, with an increasing number of potential users, would this make a significant difference for them?

 In this regard, I believe that the paper would become more engaging if the authors included a paragraph in the introduction addressing these questions.

 In conclusion, I find this manuscript to be a valuable contribution to the ongoing discussion on how to maximize the utility of opportunistic observations. I recommend its indexing after the authors have addressed the following comments.

 Comments:

1. Introduction (Page 4): The statement, "The number of opportunistic sensors has already exceeded the number of conventional instruments by an order of magnitude and is still increasing," is the only place in the manuscript where the authors explicitly mention that opportunistic measurements yield a substantial volume of data. Personally, I believe that redundancy is a key characteristic of this type of data. It would enhance the paper's appeal to readers if the authors provided a broader characterization of the available data, at least on a continental level, based on their extensive experience. Perhaps you could include a paragraph estimating the potential number of observations available for each time step and data source, offering a broad range (e.g., PWS from 10,000 to 100,000 every hour) based on the SANDBOX environment mentioned on Page 17 in your conclusions.
2. I anticipate that many readers might begin reading the paper with questions like, "How can I obtain this data?" However, the authors do not answer this question explicitly, as they make it clear from the beginning that their focus is on addressing questions such as, "Once you have the data, we propose organizing them in this manner…" To assist users, it might be helpful if you included additional references from your work within the OpenSense community that describe the process of obtaining the data as comprehensively as possible.
3. It is evident that the authors embrace a bottom-up approach, wherein individual researchers can benefit from interactions with others seeking to address similar data-related issues. Nevertheless, this approach has limitations, particularly in the identification of unique identifiers for PWS and Link Identifiers for CML and SML. What happens when different datasets are merged? Is there a way to prevent two different research groups from using the same identifier for two different PWSs, for instance? Do you have any suggestions for avoiding such situations?

Minor comments:

 Plain language summary, 1st line: Adjust the statement that includes "devices primarily intended not intended."

Where applicable, are recommendations and next steps explained clearly for others to follow? (Please consider whether others in the research community would be able to implement guidelines or recommendations and/or constructively engage in the debate)

Yes

Does the article adequately reference differing views and opinions?

Yes

Are all factual statements correct, and are statements and arguments made adequately supported by citations?

Yes

Is the rationale for the Open Letter provided in sufficient detail? (Please consider whether existing challenges in the field are outlined clearly and whether the purpose of the letter is explained)

Yes

Is the Open Letter written in accessible language? (Please consider whether all subject-specific terms, concepts and abbreviations are explained)

Yes

Reviewer Expertise:

Meteorology, climatology

I confirm that I have read this submission and believe that I have an appropriate level of expertise to confirm that it is of an acceptable scientific standard.

# Response

**Published:** 2024-01-29

**Authors:** Martin Fencl

**Affiliations:** Department of Hydrology and Hydraulics, Czech Technical Unversity in Prague, Prague 6, Czech Republic

Response to the comments *We thank both reviewers and one reader, Pinhas Alpert, for their valuable comments. We respond to the comments at respective places in the text below. Both reviews are raising questions related to availability of opportunistic sensing (OS) data. While OS data availability is intentionally not at the center of our paper, which focuses on OS data standards and formats, we understand that it is a crucial aspect for a reader (potential end user) who does not yet have access to OS data. Thus, we address the issue of OS data availability in more detail in the introducing sections of the revised version of the manuscript.* **Reviewer Report I** **Approved** Cristian Lussana, The Norwegian Meteorological Institute, Oslo, Norway The authors represent the OpenSense community, which comprises researchers and professionals primarily engaged in the public sector, including governmental institutions and universities, among others. In their paper, the authors share valuable insights into the use of opportunistic sensors for precipitation measurement. I appreciate that the authors provide concise summaries of the three data sources discussed in the paper: CML, SML, and PWS. The paper's central focus lies in recommendations regarding the typical file structure necessary for data storage. I concur with the authors' fundamental idea that adopting a common format can facilitate data sharing among OpenSense members and potentially extend to the broader scientific community. The suggested formats, both netCDF and CSV file formats, seem reasonable and well-suited for this purpose.

 However, a limitation of this paper is that it does not delve into the interaction between private companies providing the data, a scenario that frequently occurs, and the public institutions that utilize them. As a reader, I am left wondering if the proposed recommendations hold value for data providers. Do they perceive the benefits of organizing data according to the proposed structure? Moreover, with an increasing number of potential users, would this make a significant difference for them?

 In this regard, I believe that the paper would become more engaging if the authors included a paragraph in the introduction addressing these questions.

 In conclusion, I find this manuscript to be a valuable contribution to the ongoing discussion on how to maximize the utility of opportunistic observations. I recommend its indexing after the authors have addressed the following comments. *We are pleased to read that our central idea of common data standards and formats is considered valuable. Suggested OS data format recommendations evolved indeed from a bottom-up approach following the needs of OS researchers and OS data end users. We see the common data format as one important piece of the puzzle enabling the adoption of OS observations by a wide range of end users and in this sense also as an opportunity for data owners to monetize data they anyway have.* *Interoperability is a key issue when upscaling precipitation monitoring with opportunistic sensors. In this respect, standardization of data formats complying with the needs of end users plays an important role. The suggested standards evolve from OPENSENSE community having members from most of the European countries and involving eight European NHMSs and their umbrella organization EUMETNET. We are convinced OPENSENSE is able to create a critical mass required for wider acknowledgement of these standards. Designing data standards is not an isolated activity. It is part of a broader effort to boost an uptake of opportunistic sensors by relevant stakeholders in meteorology and hydrometeorology and motivate data owners to provide data for OS of precipitation and/or other atmospheric variables.* *…* Do they perceive the benefits of organizing data according to the proposed structure? Moreover, with an increasing number of potential users, would this make a significant difference for them? ... *We now partly address the potential of these data format conventions for the data owners in section in the conclusions but, at the same time, we stress there that a new format is bottom-up effort mainly initiated by OS researchers and end users associated in OpenSense network. As the OpenSense network we try reach OS data owners also at different levels, nevertheless, these efforts are out of the scope of this paper.*

 Comments:

1. Introduction (Page 4): The statement, "The number of opportunistic sensors has already exceeded the number of conventional instruments by an order of magnitude and is still increasing," is the only place in the manuscript where the authors explicitly mention that opportunistic measurements yield a substantial volume of data. Personally, I believe that redundancy is a key characteristic of this type of data. It would enhance the paper's appeal to readers if the authors provided a broader characterization of the available data, at least on a continental level, based on their extensive experience. Perhaps you could include a paragraph estimating the potential number of observations available for each time step and data source, offering a broad range (e.g., PWS from 10,000 to 100,000 every hour) based on the SANDBOX environment mentioned on Page 17 in your conclusions.

*Agreed. We have added a paragraph to Introduction estimating potential number of observations worldwide available from OS. The availability of OS data is discussed in a new subsection OS data availability.*

1. I anticipate that many readers might begin reading the paper with questions like, "How can I obtain this data?" However, the authors do not answer this question explicitly, as they make it clear from the beginning that their focus is on addressing questions such as, "Once you have the data, we propose organizing them in this manner…" To assist users, it might be helpful if you included additional references from your work within the OpenSense community that describe the process of obtaining the data as comprehensively as possible.

*We address this question in the revised manuscript in a new subsection called OS data availability. Here we refer to openly available datasets and describe some community efforts facilitating publishing of further datasets. We have also added one paragraph about data availability in the conclusions. *  

1. It is evident that the authors embrace a bottom-up approach, wherein individual researchers can benefit from interactions with others seeking to address similar data-related issues. Nevertheless, this approach has limitations, particularly in the identification of unique identifiers for PWS and Link Identifiers for CML and SML. What happens when different datasets are merged? Is there a way to prevent two different research groups from using the same identifier for two different PWSs, for instance? Do you have any suggestions for avoiding such situations?

*Thank you for pointing this out. Indeed, as a community driven by researchers we have to rely on bottom-up approach with all the limitations it brings. We cannot ensure uniqueness of identifiers globally, but they have to be unique within one dataset. We, nevertheless, agree that* *nonuniqueness of identifiers might be an issue when merging two datasets. We thus added in section Proposed data and metadata standards recommendations on merging independent datasets:*

- *We propose to mention original datasets in global attribute *reference.
- *We recommend checking possible overlapping using metadata about positions of sensors, however, we recommend to remove duplicates only if one is sure beyond a reasonable doubt that collocated sensors are the exact same data source as collocated observations (e.g. CMLs of different operators) are more common by opportunistic sensors than it is usual in traditional observational networks*
- *Unless there are specific reasons to choose otherwise, we suggest constructing new IDs, e.g. using prefixes, such as they enable user to distinguish between original data sources.*
- *We suggest describing ID taxonomy in a netCDF global attribute *comment

  Minor comments:

 Plain language summary, 1st line: Adjust the statement that includes "devices primarily intended not intended." *Corrected.*
